# Serum concentrations of afamin are elevated in patients with polycystic ovary syndrome

**DOI:** 10.1530/EC-14-0053

**Published:** 2014-06-27

**Authors:** Angela Köninger, Philippos Edimiris, Laura Koch, Antje Enekwe, Claudia Lamina, Sabine Kasimir-Bauer, Rainer Kimmig, Hans Dieplinger

**Affiliations:** 1 Department of Gynecology and Obstetrics University of Duisburg-Essen D-45122, Essen Germany; 2 Division of Genetic Epidemiology, Department of Medical Genetics, Molecular and Clinical Pharmacology Innsbruck Medical University Schöpfstrasse 41A-6020, Innsbruck Austria; 3 Vitateq Biotechnology GmbH A-6020, Innsbruck Austria

**Keywords:** vitamin E-binding protein, afamin, polycystic ovary syndrome, oxidative stress, insulin resistance

## Abstract

Oxidative stress seems to be present in patients with polycystic ovary syndrome (PCOS). The aim of this study was to evaluate the correlation between characteristics of PCOS and serum concentrations of afamin, a novel binding protein for the antioxidant vitamin E. A total of 85 patients with PCOS and 76 control subjects were investigated in a pilot cross-sectional study design between 2009 and 2013 in the University Hospital of Essen, Germany. Patients with PCOS were diagnosed according to the Rotterdam ESHRE/ASRM-sponsored PCOS Consensus Workshop Group. Afamin and diagnostic parameters of PCOS were determined at early follicular phase. Afamin concentrations were significantly higher in patients with PCOS than in controls (odds ratio (OR) for a 10 mg/ml increase in afamin=1.3, 95% CI=1.08–1.58). This difference vanished in a model adjusting for age, BMI, free testosterone index (FTI), and sex hormone-binding globulin (SHBG) (OR=1.05, 95% CI=0.80–1.38). In patients with PCOS, afamin correlated significantly with homeostatic model assessment-insulin resistance (HOMA-IR), fasting glucose, BMI, FTI, and SHBG (*P*<0.001), but in a multivariate linear model, only HOMA-IR remained significantly associated with afamin (*P*=0.001). No correlation was observed between afamin and androgens, LH, FSH, LH/FSH ratio, antral follicle count, ovarian volume, or anti-Müllerian hormone. In conclusion, elevated afamin values may indicate a state of oxidative stress and inflammation, strongly associated with IR and offering an indicator of impaired glucose tolerance in patients with PCOS irrespective of obesity.

## Introduction

The polycystic ovary syndrome (PCOS) is one of the most frequent endocrine disorders, found in up to 8% of women of reproductive age (1). According to the Rotterdam ESHRE/ASRM-sponsored PCOS Consensus Workshop Group, the diagnosis is confirmed, when two of the following three diagnostic criteria are present: i) hyperandrogenism, ii) polycystic ovaries with ≥12 sonographically measured small follicles with a diameter between 2 and 9 mm and/or an ovarian volume >10 ml, and iii) oligo- or anovulation (2).

New insights into the pathogenesis of PCOS identify oxidative stress as one possible cause, therefore offering further diagnostic and prognostic aspects (3, 4, 5, 6, 7). Oxidative stress seems to compromise follicle maturation. Surrogate parameters of oxidative stress such as ‘advanced glycosylation end products’ correlated strongly with anovulation, follicle number, and concentrations of anti-Müllerian hormone (AMH) in patients with PCOS (3).

Although insulin resistance (IR) plays an essential role in the pathogenesis of PCOS (8), it is not part of the diagnostic criteria. IR is found in 50% of women suffering from PCOS irrespective of obesity (9). *In vitro*, insulin enhances effects of luteinizing hormone (LH) in granulosa cells and leads to exaggerated androgen biosynthesis in patients with PCOS in a synergistic manner together with LH (10). An oral glucose tolerance test (OGTT) is recommended in obese patients with PCOS, but further research is required to detect lean patients with PCOS with impaired glucose metabolism (2).

In recent years, research has focused on AMH as a highly reliable diagnostic parameter for PCOS, reflecting the arrested follicle pool by acting as a follicle-stimulating hormone (FSH)-inhibiting parameter (11).

The previously described vitamin E-binding protein afamin may play a role in oxidative stress-related anti-apoptotic cellular processes (12). Vitamin E is an important anti-oxidant and protects from oxidative stress. Afamin was biochemically characterized as a vitamin E-binding protein (13) and was found in follicular fluid (14), indicating a role in the reproductive system, i.e. follicle maturation. Concentrations of afamin remain stable during the menstrual cycle (15); however, its precise role in human reproduction is virtually unknown. The afamin gene resides on chromosome 4 and was first described as the fourth member of the albumin gene family (16).

Overall, only few data exist to support an association between oxidative stress parameters and established diagnostic features of PCOS. Therefore, the purpose of our study was i) to compare serum afamin levels in patients with PCOS and healthy controls, ii) to evaluate the association between afamin and the known diagnostic criteria of PCOS including the AMH HOMA index and iii) to examine a potential diagnostic role of afamin in PCOS.

## Subjects and methods

### Study population and study design

A total of 161 women, aged 18–46 years, were consecutively enrolled in the study. Of them, 85 patients suffered from PCOS, and the remaining 76 PCOS-free patients served as controls in a cross-sectional study design. All patients were treated at the University Hospital of Essen, Department of Obstetrics and Gynecology, between 2009 and 2013. The study was conducted according to the Declaration of Helsinki for Medical Research Involving Human Patients and approved by the local research ethics committee (No. 11-4643, No. 11-4688). Informed written consent was obtained from all patients.

PCOS was diagnosed according to the Rotterdam ESHRE/ASRM-sponsored PCOS Consensus Workshop Group 2003, i.e. if two of the following criteria were present: menstrual cycle disorders (oligomenorrhea, defined as cycles lasting longer than 35 days, or amenorrhea, defined as cycles lasting longer than 3 months), clinical or biochemical signs of hyperandrogenism (hirsutism) with a Ferriman–Gallwey score of more than seven (17) or obvious acne or alopecia (18) or an elevated total testosterone (normal range 0.5–2.6 nmol/l) and/or DHEAS (normal range 6–123 μg/dl) and/or androstenedione (normal range 0.3–3.3 ng/ml), and sonographically diagnosed polycystic ovaries (at least one ovary with at least 12 follicles with a diameter of 2–9 mm each or a volume >10 ml).

Patients with endocrine disorders other than PCOS were excluded. None of the participants had taken hormonal contraceptives for at least 3 months before entering the study. Patients with tubal sterility, male causes of sterility, and patients with recurrent miscarriages but without any endocrine disorders or causes served as controls. Endocrine variables as well as afamin were determined between the second and fifth day of the menstrual cycle or after induction of artificial bleeding in cases of amenorrhea. Free testosterone index (FTI) was calculated using the formula: (total testosterone/SHBG)×100. BMI was calculated as weight/(height)^2^. In cases of elevated 17-hydroxyprogesterone, an ACTH test was performed. Cases with confirmed 21-hydroxylase deficiency were excluded from the study.

### Transvaginal scan

A 4–9-MHz transducer (Voluson E8, General Electric Systems, Vipf, Austria; IU22, Philips Healthcare, Bothell, WA, USA; Sonoline Elegra, Siemens Ultrasound Division, Munich, Germany) was used for real-time ultrasound measurements. Ovarian volume was obtained by measuring the greatest diameter in every plane. The formula for a prolate ellipsoid *V*=*x*×*y*×*z*×0.5236/1000 was used (19). For determination of the antral follicle count (AFC), small follicles with 2–9 mm diameter were calculated in the longitudinal, transverse, and anterior–posterior cross-sections of each ovary using the most available magnification factor. Women with follicles of diameter >10 mm or any kind of ovarian mass were excluded. The ovary with the most follicles and greatest ovarian volume was used for analysis.

### Glucose tolerance test

In patients with PCOS, a 3-h OGTT was used to evaluate the parameters of IR and β-cell function. After a 12-h overnight fast, patients ingested 75 g glucose and had their glucose and insulin concentrations determined at baseline and at 30, 60, 90, 120, and 180 min. For this study, we used only fasting insulin and glucose to determine the HOMA index as described previously (20).

### Biochemical analyses

Blood sample of 27 ml was collected using an S-Monovette tube (Sarstedt AG & Co., Nuembrecht, Germany) from each woman, of which 18 ml were used for hormonal analysis and 9 ml were stored at 4 °C and processed within 4 h to avoid blood cell lysis for AMH and afamin analysis. Serum was obtained by low-speed centrifugation, immediately frozen, and kept at −80 °C until analyses.

Afamin was quantified as described previously (13, 14) by a custom-made double-antibody sandwich ELISA using an affinity-purified biotinylated polyclonal anti-afamin antibody for coating 96-well streptavidin-bound microtiter plates and peroxidase-conjugated MAB N13 for detection (MicroCoat Biotechnologie GmbH, Bernried, Germany). Secondary plasma in serial dilutions initially calibrated with a primary standard served as the assay standard. Afamin purified to homogeneity from human plasma was used as the primary standard; its exact protein concentration was determined by quantitative amino acid compositional analysis. Within-run and total coefficient of variation (CV) values were 3.3 and 6.2%, respectively, at a mean concentration of 73 mg/l (15).

Automated chemiluminescence immunoassay systems were used for the determination of LH, FSH, and testosterone (ADVIA Centaur, Siemens Healthcare Diagnostics, Eschborn, Germany), androstenedione and sex hormone-binding globulin (SHBG) (Immulite 2000 XPi, Siemens Healthcare Diagnostics), and insulin (Immulite 2000 XPi, Siemens Healthcare Diagnostics). Glucose was determined photometrically (ADVIA Centaur, Siemens Healthcare Diagnostics). Intra- and inter-assay CV values for these parameters were <5 and <8% respectively.

Serum concentrations of AMH were determined by the enzymatically amplified two-site AMH-Gen-II ELISA (Beckman Coulter, Immunotech, Webster, TX, USA). Concentrations of <0.08 ng/ml were considered undetectable. Intra- and inter-assay CV values were <6%.

### Statistical methods

Median values and interquartile range (IQR) were calculated for the investigated parameters, stratified for patient and control groups. The respective distributions were compared using the Wilcoxon test. For the evaluation of afamin, Spearman's correlation coefficients were calculated with each of the parameters of interest in cases with PCOS and controls separately. In addition, a multivariate linear model was applied on afamin including all parameters that were significantly correlated with afamin at the univariate level and age. The association between afamin and PCOS case–control status was tested by means of a univariate as well as a multivariate logistic regression model, adjusted for age, BMI, FTI, and SHBG. In all regression models, skewed variables were log transformed. Statistical analyses were performed with Sigma Plot and R 3.0.

## Results

The characteristics of patients are given in Table 1. Patients with PCOS were significantly younger than controls. As expected, all tested parameters were significantly different in patients with PCOS when compared with controls. We did not assess an OGTT in the control group and consequently could not compare the parameters of β-cell function between patients with PCOS and controls. In 64 patients with PCOS, homeostatic model assessment-IR (HOMA-IR) was determined: 27 out of 64 patients had a HOMA-IR >2, indicating IR (20, 21). Out of the 85 patients with PCOS, 56 presented with severe phenotype (fulfilling all the three Rotterdam criteria) and 29 with mild phenotype (presenting with two of the three Rotterdam criteria).

Afamin concentrations were significantly higher in patients with PCOS than in controls (*P*<0.001).

In patients with PCOS, a significant positive correlation was found between afamin and BMI, fasting glucose, HOMA-IR, and FTI and a significant negative correlation between afamin levels and SHBG (*P*<0.001, Table 2). No significant correlation was observed between afamin and patient age, LH, FSH, LH/FSH ratio, testosterone, androstenedione, AFC, ovarian volume, or AMH. Subgroup analysis of patients with severe phenotype did not alter the results (data not shown). In controls, afamin was significantly and positively correlated with the BMI (*P*=0.002) and a significant inverse correlation with SHBG was also observed (*P*=0.035, Table 2).

In a multivariate linear model of afamin in patients with PCOS including BMI, age, and log-transformed values of fasting glucose, HOMA-IR, FTI, and SHBG as explanatory variables, only HOMA-IR remained significantly associated with afamin (*P*=0.001, Table 3).

A logistic regression model of afamin concerning case–control status showed a higher risk for an increase in afamin concentration (per 10 mg/l increase: odds ratio (OR)=1.307, 95% CI=1.082–1.579, *P*=0.006) being a patient with PCOS, which disappeared, however, if adjusted for age, BMI, FTI, and SHBG (per 10 mg/l increase: OR=1.050, 95% CI=0.800–1.377, *P*=0.727).

## Discussion

To our knowledge, this is the first study evaluating afamin serum concentrations in patients with PCOS and correlating them with diagnostic parameters of PCOS.

The main findings of our study are as follows:

- Afamin concentrations as well as conventional diagnostic PCOS parameters were significantly higher in patients with PCOS than in the control group.
- The difference in afamin values between patients with PCOS and controls seemed to be a consequence of obesity.
- Afamin indicated the presence of IR in patients with PCOS irrespective of obesity. As information of IR was not available in the control group, the hypothesis that it might be a consequence of IR could not be tested.

Afamin correlated with metabolic parameters such as BMI, HOMA-IR, and fasting glucose as well as with SHBG and the FTI in cases with PCOS. To evaluate which parameters were independently associated with afamin, a multiple regression analysis was performed on afamin, resulting in HOMA-IR being the only parameter that remained statistically significant. In controls, afamin showed correlations only with BMI and SHBG. No correlation was observed between serum concentrations of afamin and those of androgens, gonadotropins, sonographic PCOS parameters, or AMH. Restricting the analysis to patients with severe phenotype within our cohort did not alter the results. Nevertheless, our cohort presented with only moderate symptoms, which may result from the ethnic origin. Possibly, in a cohort with a higher degree of disease severity, e.g. more pronounced hyperandrogenism and more elevated HOMA-IR, a correlation between afamin and PCOS-specific features may occur.

We therefore conclude that IR provided the main contribution to elevated afamin concentrations in this cohort of patients with PCOS. Hence, afamin is evidence for a PCOS-associated disorder rather than for the diagnosis of PCOS, possibly indicating a state of oxidative stress and inflammation, strongly associated with IR in patients with PCOS.

Consequently, afamin is a potential candidate to indicate the need for an OGTT in patients with PCOS. Until now, OGTT was recommended only in obese patients with PCOS with a BMI ≥27 in light of the high lifetime risk for developing diabetes mellitus type 2 (2). As an OGTT with the measurement of insulin is expensive, time consuming, and restricted to experienced personnel, screening tools for lean women are strongly needed. In this context, afamin determination could detect those patients who would benefit from an OGTT. While designing the study, we did not focus mainly on biomarker search that could replace an OGTT in patients with PCOS. Therefore, our data can only be interpreted as a preliminary result possibly indicating an important biomarker of disturbed β-cell function in several, partly unknown conditions as well as in patients with PCOS.

Further research in larger cohorts is recommended to explore afamin cutoff values indicating a pathological diagnosis.

Afamin was biochemically characterized as a vitamin E-binding protein (13). Vitamin E belongs to the group of non-enzymatic antioxidants and it is plausible that its binding protein afamin indicates oxidative stress with vitamin E presence or requirement. Furthermore, there is some evidence to support an association among oxidative stress, hyperinsulinemia, and PCOS (4, 22).

Oxidative stress, defined as an imbalance between pro- and antioxidants (23), is known to be present in patients with PCOS with follicle maturation failure (3, 24). Kurdoglu *et al*. (7) demonstrated this imbalance independent of obesity and hyperinsulinemia in patients with PCOS. However, there is some evidence of an association among pathways of hyperinsulinemia, oxidative stress, and inflammation in PCOS (4, 22, 25). The authors observed a hyperglycemia-induced increase in reactive oxygen species (ROS) and activated NF-κB in patients with PCOS (4, 22), thus leading to transcription of tumor necrosis factor α (TNFα), most pronounced in obese patients with PCOS (22). Additionally, TNFα is a key marker of chronic inflammation directly inducing IR (26, 27) and strongly associated with PCOS (28). The investigation of oxidative stress status including features of metabolic syndrome in patients with PCOS and BMI-matched controls is thus highly recommended for future research.

A limitation in our study is the significant age difference between patients and controls. We have therefore adjusted our results for age although there was no significant correlation between afamin and age in our study population. This is in agreement with a previous study reporting no age dependency of afamin concentrations in a large group of healthy blood donors (15). Our control group had median afamin values of 74.9 mg/l (IQR 67.3–89.0), comparable to previously published data showing a median value of 70.7 mg/l in a healthy cohort of 177 participants without gynecological diseases (29).

## Author contribution statement

A Köninger, S Kasimir-Bauer, and H Dieplinger made substantial contributions to the study's conception and design. A Köninger, P Edimiris, L Koch, A Enekwe, R Kimmig, and H Dieplinger made substantial contributions to acquisition and interpretation of data. A Köninger, S Kasimir-Bauer, and H Dieplinger made substantial contributions to drafting the article and the final approval of the version to be published. P Edimiris., L Koch, A Enekwe, C Lamina, and R Kimmig made substantial contributions to revising the article critically for important intellectual content and the final approval of the version to be published. C Lamina gave substantial contributions to analysis and interpretation of data.

## Figures and Tables

**Table 1:** Patient characteristics, showing median (IQR) and *P* values of the Wilcoxon test.

	**Patients with PCOS**	**Controls**	***P* value**
*n*	85	76	
Age (years)	26 (23–32)	35 (30–38)	<0.001
BMI (kg/m^2^)	27.9 (23.5–35.8)	24.1 (22.0–28.4)	<0.001
Ovarian volume (ml)	13.3 (10.6–16.8)	7.7 (6.6–10.0)	<0.001
AFC	12 (10–15)	7 (5–8)	<0.001
Total testosterone (nmol/l)	2.04 (1.54–2.75)	1.32 (0.97–1.86)	<0.001
Androstenedione (ng/ml)	2.76 (2.01–3.96)	1.63 (1.17–2.03)	<0.001
FTI (%)	5.80 (3.18–9.17)	2.34 (1.54–4.32)	<0.001
SHBG (nmol/l)	41.6 (24.8–62.1)	52.7 (36.1–71.1)	<0.001
LH (IU/l)	7.4 (5.0–11.0)	4.6 (3.6–6.3)	<0.001
FSH (IU/l)	5.6 (4.2–6.9)	7.1 (6.3–8.0)	<0.001
LH/FSH ratio	1.38 (0.95–2.12)	0.64 (0.50–0.80)	<0.001
Fasting glucose (mg/dl)	87.0 (83.0–96.5)	–	–
HOMA-IR	1.32 (0.57–3.56)	–	–
AMH (ng/ml)	4.39 (2.86–7.91)	2.26 (1.05–3.00)	<0.001
Afamin (mg/l)	83.1 (72.1–101.2)	74.9 (67.3–89.0)	<0.001

AFC, antral follicle count (number of follicles with a diameter between 2 and 9 mm; FTI, free testosterone index; SHBG, sex hormone-binding globulin; LH, luteinizing hormone; FSH, follicle-stimulating hormone; HOMA-IR, homeostasis model assessment-insulin resistance; AMH, anti-Müllerian hormone.

**Table 2:** Correlation between afamin and patients' characteristics using Spearman's rank correlation test, sorted by the absolute value of the correlation coefficient for cases with PCOS.

**Parameters**	**Correlation with afamin**					
	Patients with PCOS			Controls		
*r*	*n*	*P* value	*r*	*n*	*P* value
HOMA-IR	0.65	64	<0.001	–	–	–
BMI	0.54	85	<0.001	0.34	76	0.002
Fasting glucose	0.47	67	<0.001	–	–	–
FTI	0.45	80	<0.001	0.06	52	0.693
SHBG	−0.44	81	<0.001	−0.29	52	0.035
Ovarian volume	−0.18	85	0.103	−0.01	72	0.941
Androstenedione	0.18	85	0.109	0.00	68	0.969
Testosterone	0.17	85	0.110	−0.10	75	0.387
LH/FSH ratio	−0.09	85	0.406	−0.04	74	0.763
FSH	0.09	85	0.432	−0.13	75	0.267
AFC	−0.08	85	0.461	−0.13	72	0.281
AMH	−0.07	83	0.501	−0.18	76	0.127
LH	−0.03	85	0.770	−0.12	75	0.286
Age	0.03	85	0.805	−0.06	76	0.589

**Table 3:** Results of a multivariate linear model of selected variables performed for afamin serum concentrations.

**Covariate**	**β estimate** ^a^	**95% CI** ^a^	***P* value** ^b^
HOMA-IR	2.86	0.63 to 5.09	0.001
BMI	0.49	−0.25 to 1.23	0.622
Fasting glucose	0.04	−0.23 to 0.30	0.971
FTI	0.16	−0.58 to 0.89	0.350
SHBG	−0.11	−0.28 to 0.06	0.679
Age	−0.25	−0.97 to 0.48	0.349

a Based on the original scale of variables to ensure interpretability of estimates.

b Skewed variables were log transformed (HOMA-IR, fasting glucose, FTI, and SHBG) to hold model assumptions.
